# Can lipid body-positive rates of *Mycobacterium tuberculosis* in sputum identify multidrug-resistant or rifampicin-resistant tuberculosis?

**DOI:** 10.3389/fimmu.2025.1688917

**Published:** 2025-10-15

**Authors:** Tingting Li

**Affiliations:** Changde Hospital, Xiangya School of Medicine, Central South University (The First People’s Hospital of Changde City), Changde, China

**Keywords:** *Mycobacterium tuberculosis*, high-lipid-body-positive (%LB+), nitric oxide (NO), multidrug-resistant (MDR) tuberculosis, rifampicin-resistant (RR) tuberculosis

## 1 Introduction

According to the World Health Organization (WHO), tuberculosis (TB) surpassed COVID-19 in 2023 to become the world’s leading cause of death from an infectious disease (1). This grim milestone underscores the persistent challenge of TB control, significantly compounded by the rising prevalence of multidrug-resistant (MDR-TB) and rifampicin-resistant (RR-TB) strains (1, 2). The cornerstone of effective TB management is timely and accurate diagnosis, yet the current gold standards for detecting drug resistance—molecular assays like Xpert MTB/XDR and whole-genome sequencing—remain largely inaccessible in resource-limited settings due to their high cost, technical complexity, and infrastructure requirements (2–6).

This diagnostic gap fuels transmission and compromises treatment outcomes, creating an pressing need for affordable, simple, and rapid screening tools. Ideal candidates would be techniques that can reliably identify patients at high risk of drug resistance, who could then be prioritized for confirmatory testing. This opinion focuses on one such promising candidate: the cytological detection of lipid body (LB)-positive Mtb bacilli in sputum smears and the quantification of their proportion (%LB+) (7).

The %LB+ phenotype is not a random occurrence but a well-documented physiological response of Mtb to host immune pressure, particularly nitric oxide (NO) stress (7–9). *In vitro*, exposure to NO triggers a genetic reprogramming in Mtb, upregulating genes like *tgs1* involved in triacylglycerol (TAG) synthesis, leading to the intracellular accumulation of lipid bodies (10–12). These lipid-rich bacilli enter a state of metabolic dormancy and exhibit enhanced tolerance to first-line antibiotics, effectively becoming “persister” cells (9, 13–15). Clinically, a high %LB+ in patient sputum is strongly correlated with delayed culture conversion, higher bacterial loads, and an increased risk of treatment failure and relapse (7, 10, 16).

This manuscript moves beyond a mere description of the %LB+ phenomenon. We critically explore a pivotal, unanswered question: Can the %LB+ rate serve as a phenotypic biomarker to identify Mtb strains that are not only drug-tolerant but also genotypically drug-resistant? We propose a novel synthesis of existing literature, arguing that the mechanisms driving genetic drug resistance and phenotypic tolerance through lipid body formation may be intrinsically linked. By providing a detailed explanatory framework and proposing future validation studies, this opinion aims to establish a stronger foundation for the potential clinical application of %LB+ testing in the global fight against drug-resistant TB.

## 2 The biological basis of lipid body formation: from stress response to drug tolerance

The %LB+ phenotype is a hallmark of Mtb’s adaptation to adversity. This section delves into the mechanistic link between host immune pressure, genetic regulation, and the development of a drug-tolerant state.

### 2.1 Host-induced stress as a trigger for lipid biosynthesis

The host immune response, particularly the production of nitric oxide (NO) by activated macrophages, is a key defense mechanism against Mtb. However, Mtb has evolved sophisticated countermeasures. The DosR regulon, a genetic switch activated under hypoxic and NO-rich conditions, orchestrates a transition to a non-replicating, persistent state (8). A central component of this metabolic shift is the upregulation of triacylglycerol synthase (Tgs1), catalyzing the storage of energy-rich lipid bodies (9, 11). These lipids serve as a long-term energy source, sustaining the bacterium through prolonged periods of stress and antibiotic exposure.

### 2.2 Lipid bodies: from antibiotic tolerance to clinical prognostic biomarker

This lipid-rich metabolic state is directly associated with phenotypic drug tolerance. *In vitro* studies consistently demonstrate that lipid-loaded bacilli exhibit significantly reduced susceptibility to key first-line antibiotics, particularly isoniazid and rifampicin (9, 12. 17, 12). This form of tolerance is fundamentally distinct from genetic resistance, representing instead a transient, physiological adaptation that enables bacterial subpopulations to survive antibiotic exposure without acquired mutations. Such persister populations are now widely recognized as major contributors to the requirement for prolonged therapy durations and the persistent risk of disease relapse (13, 17, 18).

Importantly, the clinical relevance of this phenotype is well established. Multiple studies have demonstrated that patients presenting with elevated %LB+ in sputum at diagnosis show an increased likelihood of poorer treatment outcomes (7, 10, 16). This correlation indicates that %LB+ effectively captures the metabolic state associated with treatment failure and relapse risk.

When considering the role of %LB+ within the broader tuberculosis diagnostic landscape, it is essential to recognize its complementary rather than competitive relationship with molecular diagnostics such as Xpert MTB/RIF (19). Molecular tests provide rapid, sensitive detection of *M. tuberculosis* complex DNA and key resistance mutations, thereby enabling prompt diagnosis and treatment initiation. In contrast, the %LB+ assay serves a distinct purpose as a functional biomarker, specifically quantifying the proportion of bacilli containing triacylglycerol-rich lipid bodies—a phenotype consistently linked to bacterial dormancy and enhanced antibiotic tolerance in experimental models (7, 20).

The clinical utility of %LB+ testing is further informed by its relationship with treatment dynamics and host immune status. Recent clinical evidence indicates that HIV co-infection correlates with significantly higher baseline %LB+ values, supporting the role of host immune status in modulating lipid body formation. Additionally, emerging research suggests that serial %LB+ measurements throughout treatment could provide valuable insights into bacterial persistence dynamics and patient-specific treatment responses (7, 21).

Therefore, the primary clinical value of %LB+ lies in its prognostic rather than diagnostic capabilities. Accumulating evidence confirms that high pretreatment %LB+ levels reliably predict unfavorable therapeutic outcomes, manifested as delayed sputum culture conversion and reduced clinical improvement (7, 16). These observations support the potential utility of %LB+ as a risk stratification tool for identifying patients at elevated risk of relapse who might benefit from intensified therapeutic approaches or extended treatment durations.

## 3 Bridging the gap: hypothesizing a link between %LB+ and genetic drug resistance

**Figure 1:**
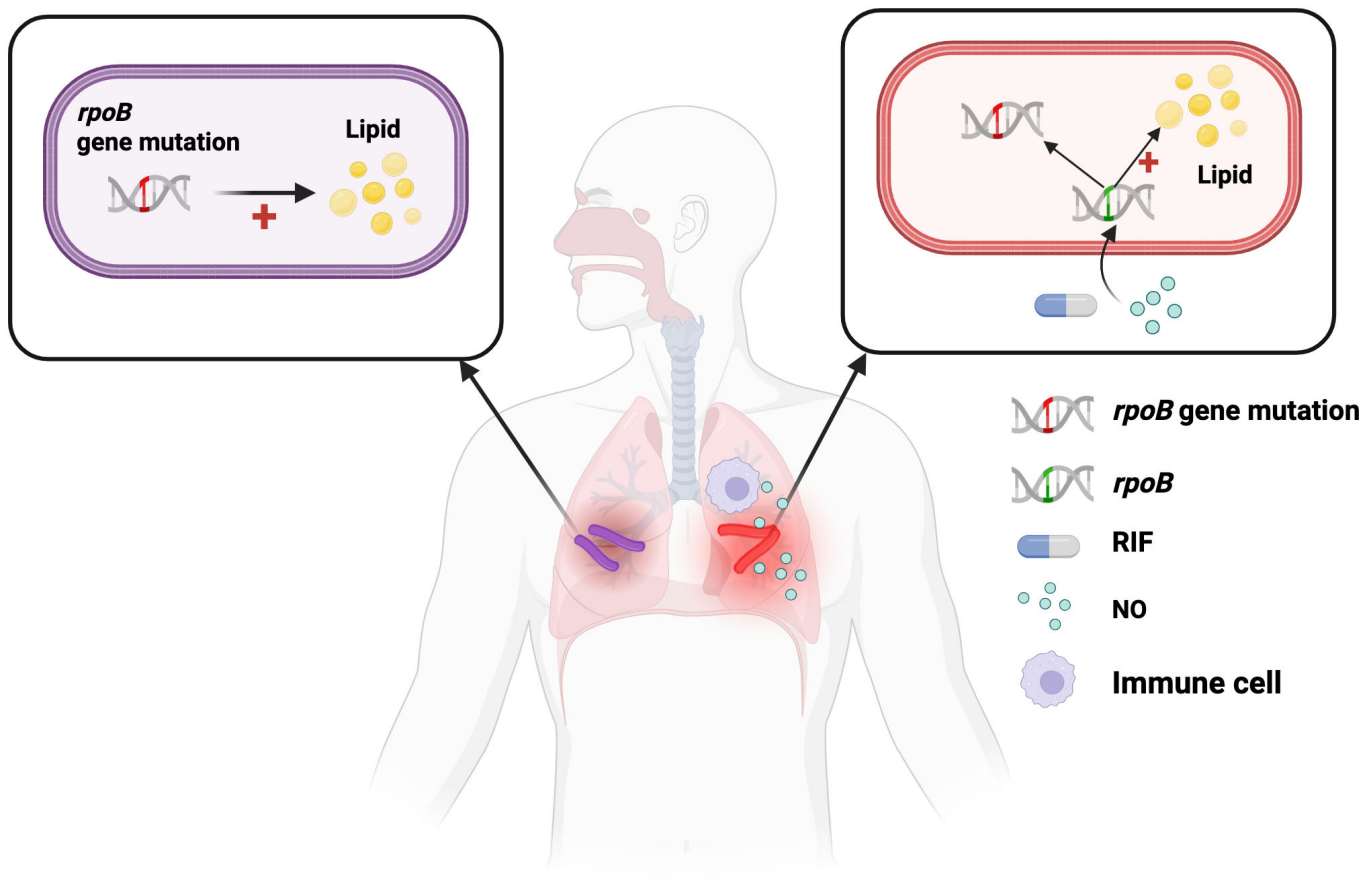
Proposed mechanisms linking elevated %LB+ with drug resistance in *M. tuberculosis*. Schematic illustration showing two proposed pathways connecting lipid body accumulation with drug resistance in *M. tuberculosis*. Left panel (Hypothesis 1): Drug resistance mutations (e.g., *rpoB*) directly promote lipid body formation as a compensatory survival mechanism. Right panel (Hypothesis 2): Host environmental conditions, including inadequate drug penetration (rifampicin, RIF) and immune stress (nitric oxide, NO), simultaneously drive drug resistance emergence and lipid body induction. Central image depicts pulmonary TB infection with cavitary lesions containing drug-resistant bacterial populations (purple and red represent different resistance mechanisms). Legend: DNA helices indicate wild-type rpoB (green) versus mutant rpoB (red); yellow circles, lipid bodies; blue capsules, rifampicin; turquoise circles, nitric oxide; purple cells, immune cells. Both pathways predict elevated %LB+ in drug-resistant strains. Created with BioRender.com(https://BioRender.com).

The critical innovation of this opinion lies in exploring the potential connection between the phenotypic %LB+ state and genotypic drug resistance. We propose two non-mutually exclusive hypotheses that could explain such a link (**Figure 1**).

### 3.1 Hypothesis 1: convergent evolution for survival

Drug resistance mutations (e.g., in *rpoB* for rifampicin resistance) confer a fitness cost to *M. tuberculosis* in the absence of antibiotics. It is plausible that strains which have evolved resistance mutations are also under selective pressure to acquire compensatory mechanisms that enhance their general resilience. The ability to rapidly induce lipid body formation in response to stress could be one such mechanism (**Figure 1**, left panel). In this model, drug-resistant bacilli carrying mutations such as *rpoB* directly upregulate lipid metabolism pathways, leading to enhanced lipid body accumulation. Therefore, drug-resistant strains may be predisposed to exhibit a higher baseline or inducible %LB+ compared to pan-susceptible strains as a general survival advantage. This represents a direct genetic link between resistance mutations and the %LB+ phenotype.

### 3.2 Hypothesis 2: common inducing conditions

Alternatively, the conditions that favor the emergence of drug resistance—inadequate drug exposure (e.g., poor rifampicin penetration into cavitary lesions), compromised immune control, and cavitary disease—are the same conditions that generate high immune stress and induce the %LB+ phenotype (**Figure 1**, right panel). Within the hostile microenvironment of pulmonary cavities, bacilli encounter multiple stressors including reactive nitrogen intermediates (e.g., NO) produced by immune cells, suboptimal drug concentrations, and nutrient limitation (20, 22, 23). These environmental pressures simultaneously drive two adaptive responses: selection for drug resistance mutations and induction of metabolic dormancy characterized by lipid body formation.

In this scenario, a high %LB+ may not be a direct result of the resistance mutation itself but rather a marker of the dysfunctional host-pathogen interaction that creates permissive conditions for both phenotypes to emerge and persist. Thus, %LB+ would act as an indirect biomarker, identifying a patient ecology characterized by inadequate bacterial clearance, suboptimal drug penetration, and sustained immune activation—conditions that are permissive for both drug tolerance and resistance development.

A preliminary study by Tarekegn et al. supports the feasibility of this investigation, showing significant variability in %LB+ among patient samples and correlating this variability with both host immune markers and bacterial factors (7). The next logical step is to analyze whether this variability is stratified by the drug resistance profile of the infecting strain.

## 4 Detailed methodology of %LB+ quantification and associated technical considerations

The accurate quantification of %LB+ employs a standardized multiparametric approach combining fluorescence staining with automated image analysis. The established protocol, as documented in methodological studies, involves sequential processing stages each requiring precise standardization (7, 11).

### 4.1 Sample processing and staining protocol

The analytical process initiates with standardized sputum processing using N-acetyl-L-cysteine-sodium hydroxide (NALC-NaOH) decontamination and concentration. Critical to lipid preservation is vapor-phase formaldehyde fixation of prepared smears, which maintains both cellular morphology and lipid body integrity. The core staining methodology employs sequential fluorescence applications: primary staining with Auramine O for acid-fast bacilli identification, followed by secondary staining with neutral lipid-specific dyes—either Nile Red (10 µg/mL in PBS) or LipidTox Red (1:200 dilution in PBS)—with a standardized 10-minute incubation period. Epifluorescence microscopy utilizes distinct filter configurations: FITC filters (excitation 460/25 nm, emission 550/25 nm) for Auramine O visualization and TRITC filters (excitation 560/20 nm, emission 630/37.5 nm) for lipid dye detection. The sequential imaging must be completed within a single session to prevent fluorescence decay and ensure precise signal colocalization.

### 4.2 Image analysis and quantification

The transformation of fluorescent signals into quantitative %LB+ data represents the most technically demanding phase. Automated analysis platforms, typically employing customized ImageJ algorithms or similar computational approaches, execute several critical functions: application of rolling-ball background subtraction (50-pixel radius) to correct illumination heterogeneity, individual bacterial segmentation based on Auramine O signal characteristics, and corresponding lipid-channel fluorescence intensity measurement for each bacillus. Classification as lipid-body-positive requires fluorescence intensity exceeding predetermined thresholds established through control samples. Analytical rigor necessitates examination of ≥20 microscopic fields encompassing >200 individual bacilli to ensure statistical reliability.

### 4.3 Technical challenges and standardization issues

Several technical challenges complicate %LB+ quantification and require careful standardization:

#### 4.3.1 Pre-analytical variability

Specimen heterogeneity and differences in decontamination efficiency can significantly impact lipid body preservation. Bacterial culture methods, particularly those involving centrifugation stress, may artificially induce lipid body formation.

#### 4.3.2 Staining consistency

Inter-batch variations in dye potency, concentration accuracy, incubation timing, and temperature control introduce measurement variability. The differential performance characteristics between Nile Red and LipidTox Red—including binding affinity, photostability, and fluorescence quantum yield—complicate cross-study comparisons.

#### 4.3.3 Imaging standardization

Inconsistent microscope calibration, including variations in light source intensity, camera sensitivity settings, and filter performance, can substantially affect fluorescence measurements. The lack of standardized reference materials for instrument calibration remains a significant limitation.

#### 4.3.4 Analytical threshold determination

Objective establishment of fluorescence intensity thresholds for positive classification presents considerable challenges. Current approaches rely on laboratory-specific controls, creating inter-laboratory variability.

#### 4.3.5 Sample representativeness

The inherent heterogeneity of sputum samples necessitates comprehensive sampling strategies. Inadequate field selection or insufficient bacillary counts may yield non-representative %LB+ estimates.

### 4.4 Standardization initiatives and future directions

Addressing these challenges requires implementation of rigorous standardization protocols including:

- Development of certified reference materials for instrument calibration
- Establishment of standardized operating procedures for staining and imaging
- Implementation of external quality assessment programs
- Transition toward automated, high-content imaging systems to reduce operator-dependent variability
- Multi-laboratory validation studies to establish consensus thresholds

These methodological considerations highlight both the current capabilities and limitations of %LB+ quantification while outlining pathways toward enhanced reproducibility and clinical utility.

## 5 Clinical implications and a proposed diagnostic workflow

**Figure 2:**
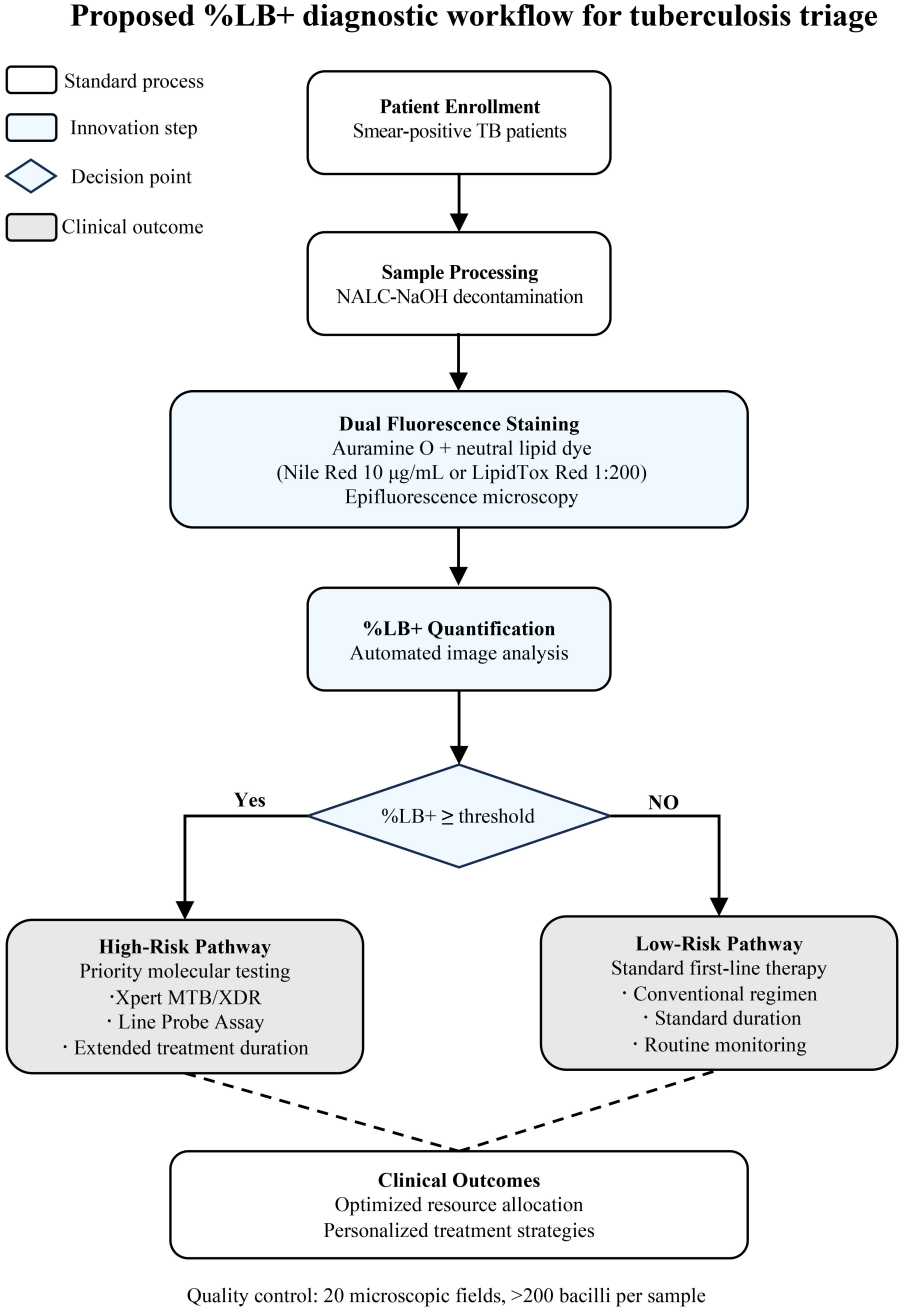
Proposed %LB+ diagnostic workflow for tuberculosis triage. Schematic illustration of a risk stratification algorithm for smear-positive TB patients. Following NALC-NaOH decontamination, sputum samples undergo dual fluorescence staining (auramine-O + neutral lipid dye: Nile Red 10 μg/mL or LipidTox Red 1:200). Automated image analysis quantifies %LB+ via epifluorescence microscopy (quality control: ≥on fields, >200 bacilli). High %LB+ patients (atients.yce receive priority molecular testing (Xpert MTB/XDR, line probe assay) and extended treatment; low %LB+ patients (<threshold) proceed to standard first-line therapy. Grey boxes, standard processes; blue boxes, innovation steps; diamond, decision point; dashed lines, pathways to clinical outcomes. This triage approach optimizes molecular diagnostic resources while enabling personalized treatment based on bacterial metabolic state.

Upon validation, the %LB+ assay could be integrated into a cost-effective diagnostic algorithm for resource-constrained settings. **Figure 2** illustrates the proposed workflow for using %LB+ as a triage tool to optimize molecular test allocation.

### 5.1 Workflow overview

The diagnostic algorithm (**Figure 2**) begins with standard sample processing and dual fluorescence staining of sputum from smear-positive TB patients. Following automated %LB+ quantification, patients are stratified based on predetermined thresholds: those with high %LB+ receive priority molecular testing (Xpert MTB/XDR or line probe assay) and may benefit from extended treatment, while those with low %LB+ proceed directly to standard first-line therapy.

### 5.2 Clinical advantages

This triage approach offers several benefits: (1) Resource optimization through selective use of expensive molecular tests; (2) Improved outcomes via faster identification and treatment of drug-resistant cases; (3) Personalized treatment guided by bacterial metabolic state; and (4) Scalability using existing microscopy infrastructure with minimal training requirements.

### 5.3 Path forward

Implementation requires validation of optimal thresholds across diverse populations and integration with existing tuberculosis diagnostic guidelines. This approach represents a practical solution for enhancing diagnostic accuracy while addressing resource constraints in high-burden settings.

## 6 Conclusion and future directions

The %LB+ assay represents a unique opportunity to leverage a simple phenotypic observation into a powerful tool for managing drug-resistant TB. This opinion has argued that a connection between lipid body content and drug resistance is biologically plausible and clinically significant. However, moving from concept to practice requires rigorous validation.

We propose the following research agenda:

### 6.1 Retrospective cohort studies

Re-analyze stored sputum samples from well-characterized cohorts of drug-susceptible, MDR-, and RR-TB patients. Quantify the %LB+ in a blinded manner and perform statistical analyses to determine if significant differences exist between groups.

### 6.2 *In vitro* modeling

Expose isogenic pairs of susceptible and resistant Mtb strains to various stresses (NO, antibiotics, hypoxia) and compare the kinetics and magnitude of lipid body induction and drug tolerance.

### 6.3 Prospective validation

Design a prospective study in a high-burden setting to validate the diagnostic accuracy (sensitivity, specificity, PPV, NPV) of the %LB+ threshold for predicting MDR/RR-TB.

By undertaking these studies, the scientific community can determine whether this inexpensive and accessible assay can truly become a frontline defense in the escalating battle against drug-resistant tuberculosis.
